# Gene expression profiling of noninvasive primary urothelial tumours using microarrays

**DOI:** 10.1038/sj.bjc.6602813

**Published:** 2005-11-01

**Authors:** M Aaboe, N Marcussen, K M-E Jensen, T Thykjaer, L Dyrskjøt, T F Ørntoft

**Affiliations:** 1 Molecular Diagnostic Laboratory, Department of Clinical Biochemistry, Aarhus University Hospital Skejby, 8200 Aarhus N, Denmark; 2 Institute of Pathology, Aarhus University Hospital, Aarhus Hospital, 8000 Aarhus C, Denmark; 3 Department of Urology, Aarhus University Hospital Skejby, 8200 Aarhus N, Denmark

**Keywords:** bladder cancer, gene expression, DNA microarray, keratin

## Abstract

At present, the mechanism leading to bladder cancer is still poorly understood, and our knowledge about early events in tumorigenesis is limited. This study describes the changes in gene expression occurring during the neoplastic transition from normal bladder urothelium to primary Ta tumours. Using DNA microarrays, we identified novel differentially expressed genes in Ta tumours compared to normal bladder, and genes that were altered in high-grade tumours. Among the mostly changed genes between normal bladder and Ta tumours, we found genes related to the cytoskeleton (keratin 7 and syndecan 1), and transcription (high mobility group AT-hook 1). Altered genes in high-grade tumours were related to cell cycle (cyclin-dependent kinase 4) and transcription (jun d proto-oncogene). Furthermore, we showed the presence of high keratin 7 transcript expression in bladder cancer, and Western blotting analysis revealed three major molecular isoforms of keratin 7 in the tissues. These could be detected in urine sediments from bladder tumour patients.

Bladder cancer is a serious health problem. Neoplastic transformation of urothelial cells gives rise to urothelial carcinomas, which has a very high prevalence in Western societies (Edwards *et al*, 2002). Patients diagnosed with bladder cancer often get recurrent tumours. Tumour recurrence develops in 35% of patients with papillary urothelial neoplasm of low malignant potential (PUNLMP), in 71% of patients with low-grade urothelial carcinoma, and in 73% of patients with high-grade carcinoma (Holmang *et al*, 2001). Ta tumours are superficial papillary neoplasms that do not invade the submucosa and muscle layers of the bladder. At present, the mechanism leading to bladder cancer is still poorly understood, and our knowledge about early changes occurring during transformation of normal urothelium into noninvasive Ta tumours is limited. Over recent years, much work has been carried out to reveal events relevant to understanding the aetiology of bladder cancer. It has become apparent that urothelial carcinoma is developing through a multistep process with increasing accumulation of multiple genetic alterations (reviewed in Orntoft and Wolf, 1998). Previous studies have shown the importance of genetic alterations affecting known oncogenes and tumour suppressor genes (Knowles, 1999). An increase in grade, characterised by a loss of differentiation, and the presence of dysplasia as well as tumour multiplicity are determinants of poor prognosis (Heney, 1992; Prout *et al*, 1992). Studies using microsatellites, and more recently, array-based comparative genomic hybridisation, have shown that loss of heterozygosity (LOH) especially loss of chromosome 9 is a frequent finding in early stage bladder cancer (Cairns *et al*, 1993; Ruppert *et al*, 1993; Keen and Knowles, 1994; Primdahl *et al*, 2002). Many molecular variables have been suggested to be useful in the classification and prediction of progression. Among those have been oncogenes (H-ras), tumour suppressors (p53), cell-cycle proteins, growth factors and cell adhesion molecules to name a few (Cordon-Cardo *et al*, 2000; Oxford and Theodorescu, 2003).

DNA microarray analysis has emerged as an important tool to identify genes that are differentially expressed in normal and tumour tissues. Previously, we have used this technology to identify classes of differentially expressed genes by which stage Ta tumours could be separated from muscle-invasive stage T2-4 tumours (Thykjaer *et al*, 2001), and for carcinoma *in situ* (Dyrskjot *et al*, 2004). Furthermore, in a recent study, we identified unique gene expression profiles for the three major clinical stages of bladder cancer, Ta, T1 and T2-4 (Dyrskjot *et al*, 2003). In the present study, we focus on the neoplastic transition from normal bladder urothelium to primary Ta tumours. Using DNA microarrays, we define the early gene expression changes that occur in urothelial neoplasia. We identify novel differentially expressed genes in Ta tumours compared to normal bladder, and genes that are altered in high-grade tumours. Four of these (KRT7, SDC1, CDK4 and JUND) are further characterised with respect to location by immunohistochemistry. In addition, we show that keratin 7 is highly expressed in bladder cancer and is correlating to the protein level, can be detected in urine sediments, and has three major molecular isoforms, as determined by Western blotting.

## MATERIALS AND METHODS

### Tissue material

Urinary bladder tumour specimens (stage Ta) were obtained after removal of the necessary amount of tissue for routine pathology examination. Normal bladder tissue biopsies were obtained from individuals with no history of bladder tumours. They were removed by taking a cold cup biopsy, to preserve RNA and to maximise the amount of normal urothelium. Tissue samples were frozen immediately after surgery and stored at −80°C in a RNA preserving solution of guanidinium thiocyanate. Samples were evaluated twice according to WHO/ISUP (Epstein *et al*, 1998). A training data set was defined containing 34 samples; 25 primary Ta tumours (10 PUNLMP, five low grade, and 10 high grade) and nine normal bladder biopsies. The nine normal bladder biopsies were also included in an evaluation data set 1 (*N*=32), which contained another 23 Ta tumours (11 PUNLMP, two low grade, and 10 high grade). A third data set (*N*=81) was defined, which contained the previously described normal (9) and Ta (48) samples, as well as 24 additional muscle-invasive T2-4 tumours (evaluation data set 2). Tissue for immunohistochemistry was formalin-fixed and embedded in paraffin. Informed consent was obtained in all cases, and protocols were approved by the local scientific ethical committee.

### Target preparation

Total RNA was purified from tissue samples using a FastPrep® instrument and FastRNA® Kit-Green tubes (Qbiogene, Irvine, USA), followed by the RNeasy® mini kit isolation method (Qiagen, Venlo, NL). From 10 *μ*g of total RNA double-stranded cDNA was prepared using the SuperScript Choice System (Invitrogen, Carlsbad, USA), according to the manufacturer's instructions, except that we used an oligo-dT primer containing a T7 RNA polymerase promoter site (DNA Technology A/S, Aarhus, DK), as described by Affymetrix. Labelled cRNA was prepared using the BioArray High Yield RNA Transcript Labeling kit (Enzo Biochem, Farmingdale, USA). Biotin-labelled CTP and UTP (Enzo Biochem, Farmingdale, USA) were used in the reaction, together with unlabelled nucleotide triphosphates. Unincorporated nucleotides were removed from the *in vitro* transcript using the RNeasy® mini kit isolation method (Qiagen, Venlo, NL).

### DNA microarray hybridisation, washing, and scanning

We fragmented 15 *μ*g of cRNA at 94°C for 35 min in a fragmentation buffer containing 40 mM Tris-acetate (pH 8.1), 100 mM potassium acetate and 30 mM magnesium acetate. Prior to hybridisation, the labelled cRNA mixed with hybridisation buffer (0.1 M MES, 1 M NaCl, 20 mM EDTA, 0.01% Tween-20) was preheated in 5 min to 99°C and then cooled to 45°C for 5 min before loading onto the Affymetrix microarray (HG-U133A). We then incubated the probe array for 16 h at 45°C at constant rotation (60 r.p.m.). The washing and staining procedure was performed in the Affymetrix Fluidics Station. We exposed each microarray to 10 washing cycles using nonstringent wash buffer (6 × SSPE, 0.01% Tween-20) at 25°C followed by four rounds of wash in stringent wash buffer (100 mM MES, 0.1 M NaCl, 0.01% Tween-20 at 50°C. Signal intensities were increased by antibody amplification. First the microarray was stained in SAPE stain solution (1 × MES buffer, 2 mg ml^−1^ BSA, 10 *μ*g ml^−1^ Streptividin-Phycoerythin (SAPE)), washed in nonstringent wash buffer, stained in antibody solution (1 × MES, 1 M NaCl, 0.05% Tween-20, 2 mg ml^−1^ acetylated BSA, 100 *μ*g ml^−1^ normal goat IgG, 3 *μ*g ml^−1^ biotinylated antibody), stained with SAPE stain solution again, and finally washed in nonstringent wash buffer. Microarrays were then scanned at 560 nm using a confocal laser-scanning microscope with an argon ion laser as the excitation source (GeneArray Scanner G2500A; Hewlett Packard, Palo Alto, USA).

### DNA microarray data analysis

Microarray data were normalised and probe expression measures calculated using the robust multiarray analysis normalisation method (Bolstad *et al*, 2003) implemented in the ArrayAssist 3.0 software package (Stratagene, La Jolla, USA). Nonvarying genes were eliminated by filtering out genes with variation less than 8000 across the microarrays used. Student's *t*-test was applied for comparisons between independent groups, that is training data set, (a) primary Ta tumours *vs* normal bladder biopsies and (b) primary Ta high-grade tumours *vs* primary Ta PUNLMP/low grade; evaluation data set 1, (a) Ta tumours *vs* normal bladder biopsies; and evaluation data set 2, normal bladder biopsies *vs* Ta and T2-4 tumours. *P*-values were adjusted for multiplicity using the Benjamini–Hochberg correction procedure (Benjamini and Hochberg, 1995). We defined as candidate informative genes those with unadjusted *P*<0.05, and as differentially expressed genes those with a significant expression difference (adjusted for multiplicity *P*<0.05).

### Identification of over-represented gene categories

Genes can be placed in functional categories, and it is then possible to test if altered genes are primarily belonging to certain functional groups. Over-represented gene categories were identified using EASE software (Hosack *et al*, 2003). For both the candidate informative genes (comparing Ta high-grade and PUNLMP/low-grade tumours) and the differentially expressed genes (comparing normal and Ta tumours), we determined the major biological themes. All significant (*P*<0.05) gene categories were determined by EASE score, corrected for multiplicity with the global false-discovery rate function using 1000 iteration trials.

### Cluster analysis

Unsupervised hierarchical cluster analysis was performed using Cluster software. For visualisation we used TreeView software (Eisen *et al*, 1998). Nonvarying probesets were eliminated from the training data set by filtering away probesets with standard deviation less than 0.5, and expression levels less than 6.5 in at least four microarrays (log 2-transformed data). The filtering process gave 4144 varying probesets. Microarrays were median-centred, normalised to the magnitude of 1, and clustered using average linkage clustering with a modified Pearson correlation as similarity metric.

### Immunohistochemistry

Paraffin-embedded tissue sections were deparaffinised, rehydrated, boiled in a microwave oven for 5 min, allowed to cool at room temperature for 20 min, and then incubated in methanol/H_2_O_2_ (100 ml methanol+1.5 ml w v^−1^ H_2_O_2_) for 10 min to block endogenous peroxidase. The tissue sections were then incubated in TEG buffer (Tris-EGTA, pH 9.0) boiled for 10 min in a microwave, allowed to cool at room temperature for 20 min, incubated with Pronase (DakoCytomation A/S, Glostrup, DK) for 20 min at room temperature. Then tissue sections were incubated with specific antibodies against KRT7(sc-23876) (1 : 1200), SDC1(sc-12765) (1 : 100), CDK4(sc-749) (1 : 50) and JUND(sc-74) (1 : 50) all purchased from Santa Cruz Biotechnology. The primary antibodies were visualised with the Envision™ visualisation system and the chromogen DAB+ from DakoCytomation A/S (K3868), followed by counterstaining with Mayer's haematoxylin and mounting in Aquatex® (1.08562; Merck, Darmstadt, Germany). Immunohistochemical (IHC) evaluation was carried out by two independent investigators (LD and MA), who scored the cellular localisation of protein expression. In case of disagreement a consensus evaluation score was achieved.

### Western blotting

Fresh protein extractions were prepared using RIPA lysisbuffer (50 mM Tris pH 7.5, 150 mM NaCl, 1% NP-40, 0.5% deoxycholic acid) with protease inhibitors added (Complete protease Inhibitor Cocktail Tablet, Roche, Basel, CH). Approx. 15 mg frozen tissue was mixed with ice cold lysisbuffer (100 *μ*l per 5 mg tissue) using the FastPrep instrument (Qbiogene, Irvine, USA) for 2 × 20 s to release the total proteins. Contaminating urea and salt was removed from urine and urine pellet samples by o/n dialysis against dH_2_O (4°C) using Spectra/Por membrane (Spectrum Chromatography, Houston, USA). Samples were freeze-dried and lysisbuffer was added. Lysates were mixed thoroughly and incubated on ice for 30 min and followed by centrifugation (13 000 **g** for 10 min at 4°C). Protein concentrations were determined by Bradford protein assay (Bio-Rad, Hercules, USA). SDS–PAGE was performed under reducing conditions at 200 V for 50 min (10 *μ*g total protein per lane). Proteins were transferred to a methanol activated PVDF membrane (Millipore, Billerica, USA) by blotting at 30 V for 2 h. Before incubating with antibody, the membrane was blocked in blocking buffer (1 × phosphate-buffered saline (PBS), 0.1% Tween-20, 5% skim milk) for 30 min at RT. We then incubated with anti-KRT7 antibody (1 : 2000) (sc-23876; Santa Cruz Biotechnology, Santa Cruz, USA) for 1 h at RT, washed three times in washing buffer (1 × PBS, 0.1% Tween-20), and incubated with goat anti-mouse HRP (1 : 5000) (P0447; DakoCytomation A/S, Glostrup, DK) secondary antibody for 1 h at RT. The immunoreactive complex was visualised by the ECL chemiluminescent substrate (GE healthcare, Chalfont St Giles, UK) and detected by STORM imaging system (GE healthcare, Chalfont St Giles, UK). For quantification, we used the ImageQuant 5.0 software (GE healthcare, Chalfont St Giles, UK).

### Statistics

The *χ*^2^ test was used to assess the relation between tumour grade and clustering pattern. Differences in median recurrence time between the PUNLMP and high-grade subcluster groups were assessed using Kaplan–Meier survival analysis, the statistical significance was assessed by the log-rank test for equality of survivor functions. The analysis was based on all follow-up information recorded until March 1, 2004. Values of *P*<0.05 were considered statistically significant. Intercooled Stata 8 software (StataCorp LP, College Station, USA) was used throughout.

## RESULTS

### Genes differentially expressed between normal bladder and primary Ta tumours

In order to identify gene expression changes of relevance for early bladder transformation, the transcriptional profiles of 25 primary Ta tumours from bladder cancer patients were compared with those of nine normal bladder biopsies from patients with no bladder cancer history. Total RNA was extracted from tissue biopsies, and DNA microarrays (Affymetrix HG-U133A) comprised of more than 22 000 probe sets were used to analyse gene expression profiles. Nonvarying genes were eliminated by filtering out genes with low variance. A panel of 230 differentially expressed genes (*P*<0.00005) out of 2416 varying genes distinguished the Ta tumours from normal biopsies (Supplementary Table 1). Of those, 228 (99.1%) were upregulated in Ta tumour relative to normal biopsies and only two were downregulated. Table 1 shows the most significant genes identified, some of which have already been linked to neoplasia. Genes related to *cytoskeleton* were keratin 7 (KRT7), keratin 8 (KRT8), junction plakoglobin (JUP), and syndecan 1 (SDC1); to *transcription*, high mobility group AT-hook 1 (HMGA1); to *protein folding*, fatty acid binding protein 6, ileal (gastrotropin) (FABP6) and G-rich RNA sequence binding factor 1 (GRSF1); and to *transferase activity*, CD24 antigen (small-cell lung carcinoma cluster 4 antigen) (CD24) and lamin A/C (LMNA). For validation, we repeated the comparison in a set of 23 independent Ta tumour samples and confirmed the expression profiles of, for example, KRT7 (adjusted for multiplicity *P*<0.05) and SDC1 (adjusted for multiplicity *P*<0.05) in this data set (evaluation data set 1). In total, we were able to validate 228 of 230 genes this way. The two genes that were not validated are marked with an asterisk in Supplementary Table 1.

### Altered gene expression in tumours with high-grade atypia

Cancer cells from high-grade tumours contain distinct characteristics as they in contrast to low-grade tumours frequently display abnormal cell size, shape, and large hyperchromatic nuclei, and are associated with a poorer outcome. To identify gene expression changes related to the high-grade phenotype, we made a comparative analysis between primary Ta high-grade tumours and Ta PUNLMP and low-grade tumours (*N*=25). Nonvarying genes were eliminated as previously described. We obtained a panel of 86 candidate informative genes (*P*<0.005) out of 2316 varying genes that distinguished high-grade tumours from PUNLMP/low-grade tumours (Supplementary Table 2). Similar to previous findings, we also in this case found most genes upregulated (87.2%) in high-grade tumours compared to PUNLMP/low-grade tumours. The genes that contributes to the molecular signature of high-grade tumours are, not unexpected, related to regulation of the *cell cycle*, cyclin-dependent kinase 4 (cdk4); regulation of *cell growth*, hypothetical protein MAC30 (MAC30); and *transcription*, interleukin enhancer binding factor 2, 45 kDa (ILF2), formin binding protein 3 (FNBP3), and jun D proto-oncogene (JUND) (Table 2). The changes in gene expression of individual genes were lower when looking at specific grades in Ta tumours compared to normal biopsies. This could reflect a biological phenomenon and makes it more difficult to find highly significantly changing genes when comparing grades, unless using very large samples sets. The candidates selected seemed relevant based on their purported function.

### Hierarchical clustering of primary Ta tumours

**Figure 1:**
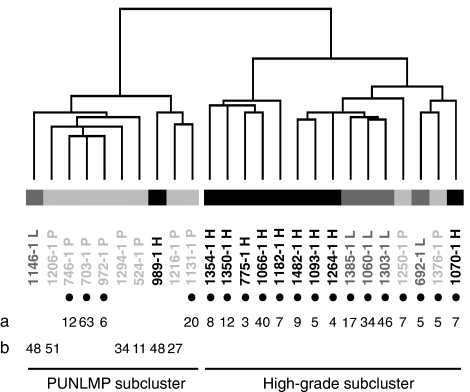
Unsupervised hierarchical cluster analysis of 25 Ta tumours using 4144 genes. Tumour recurrences are represented by black dots. (a) Time to recurrence in months, and (b) follow-up time in months. Abbreviations: Ta PUNLMP (Ta P; coloured light grey), Ta low-grade (Ta L; coloured grey), Ta high-grade (Ta H; coloured black).

**Figure 2:**
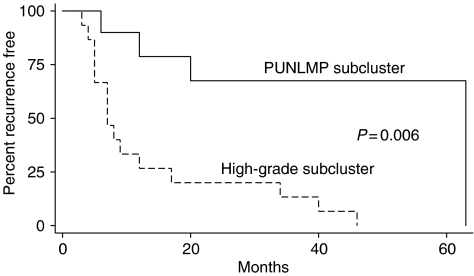
Kaplan–Meier survival analysis of the median time to recurrence in the PUNLMP subcluster and the high-grade subcluster.

Unsupervised cluster analysis was performed using the 25 primary Ta tumours to study the possible relationship between gene expression patterns and cellular atypia grading. The clustering identified two groups of Ta tumours, which clearly differed in grades of atypia (Figure 1). One subcluster contained primarily PUNLMP tumours (denoted PUNLMP subcluster), and the second had a high content of high-grade tumours (denoted high-grade subcluster). The distribution of PUNLMP and high-grade tumours was significant using *χ*^2^ test (*P*=0.009). Low-grade tumours were found primarily to cluster with the high-grade tumours. We estimated the median time to recurrence using Kaplan–Meier survival statistics. The median time to recurrence of high-grade subcluster tumours was, as expected, decreased compared to PUNLMP subcluster tumours; 7 months and 63 months, respectively (*P*=0.006) (Figure 2). However, the low-grade tumours seemed to have the same recurrence pattern as the high grade or PUNLMP tumours that they clustered together with.

### Identification of over-represented gene categories

Identification of over-represented gene categories in gene lists is a powerful tool to identify possible biological themes involved. We applied the EASE software (Hosack *et al*, 2003) to search for over-represented gene categories in the two lists of genes identified as showing an altered expressed between primary Ta tumours and normal biopsies (230 genes), and between primary Ta high-grade tumours and primary Ta PUNLMP/low-grade tumours (86 genes). EASE performs a comparative analysis between gene categories represented by single gene lists, and gene categories represented by all genes found on the microarray. The Affymetrix HG-U133A microarray contains 10937 genes annotated within the biological process branch of the Gene Ontology (GO) (Ashburner *et al*, 2000). Approximately 73% (168 genes) of the genes found differentially expressed in Ta tumours compared to normal bladder biopsies were annotated. Several over-represented gene categories were identified, mostly related to an increased energy turn over, for example, protein folding, mRNA splicing, energy pathways, glucose catabolism, lipid metabolism (Table 3). The analysis was repeated for the panel of 86 candidate informative genes that distinguished high-grade tumours from PUNLMP/low-grade tumours. In this case, 67 out of 86 genes were annotated. The over-represented gene categories were related to RNA/DNA metabolism and proliferation such as RNA processing, RNA metabolism, protein-nucleus import, and regulation of cell cycle (Table 3).

### Immunohistochemistry

**Figure 3:**
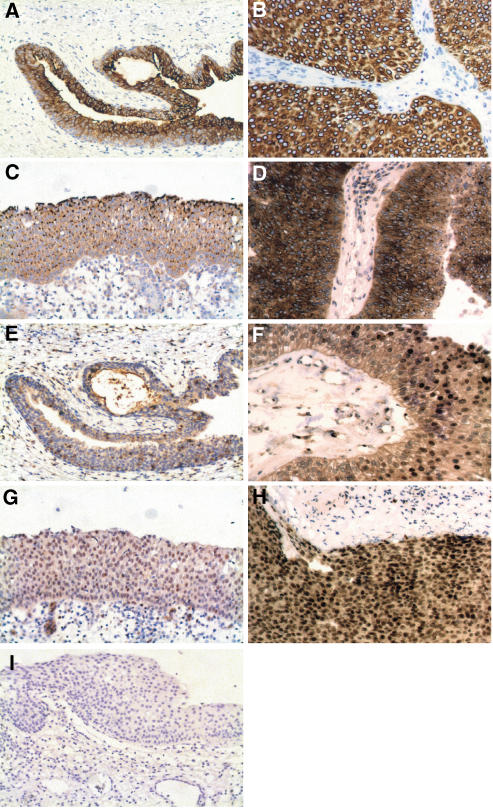
Representative immunohistochemical stains for (**A**, **B**) KRT7, (**C**, **D**) SDC1, (**E**, **F**) CDK4 and (**G**, **H**) JUND in Ta tumours in normal urothelium (left panel) and Ta tumour (right panel). No ab control (**I**). Original magnification: × 20.

We used IHC staining to verify that the mRNA was being translated into protein, and to document the cellular localisation of the proteins. The proteins encoded by a subset of four genes were analysed. They were selected to represent highly significantly changed genes, more moderately changed candidate genes, and based on the availability of commercial antibodies. Two had an expected localisation within the cytoplasma (KRT7) or the nucleus (CDK4), and two with no reports on protein localisation in urothelium (SDC1 and JUND). We have included the results from the complete evaluation in Supplementary Table 3. In concordance with previously reports (Glass and Fuchs, 1988), KRT7 was completely absent in stroma cells, but cytoplasmic expressed in most normal urothelial (85%) and all cancer cells (100%). We did not observe any nucleic stainings for KRT7. In agreement with the gene expression profile of CDK4, its cytoplasmic staining was increasing from 20% in normal urothelial cells to 100% of cancer cells. The nucleic CDK4 staining was also increasing from 5% in normal urothelial cells to 50% in cancer cells (Figure 3). The cell surface proteoglycan protein SDC1 link the cytoskeleton to the interstitial matrix (Bernfield *et al*, 1999). SDC1 displayed a spotted cytoplasmic staining pattern in cancer cells often with an accentuation close to the nucleus. We observed an increase in cytoplasmic SDC1 staining in cancer cells (100%) compared to normal urothelial cells (65%). Previously, reports has described SDC1 protein expression linked to the cell membrane, and loss of membrane staining has been reported to correlate with poor prognosis (Harada *et al*, 2003). The observed increase of JUND gene expression in tumours was accompanied by increased protein expression. JUND protein expression was increased in cancer cells compared to normal urothelial cells. The percentage of stained nuclei increased from 55 to 90%, and the cytoplasmic stain increase from 65 to 100% in cancer cells.

### Keratin 7 expression in bladder cancer

**Figure 4:**
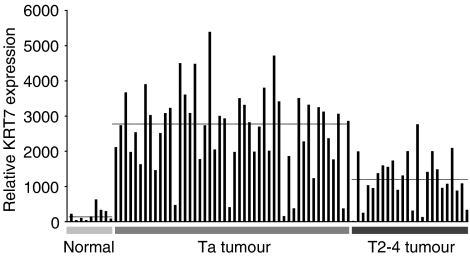
Expression of KRT7 transcript in normal bladder biopsies, Ta, and T2-4 tumours (*N*=81) measured by microarray analysis. Median expression levels for each group are represented by horizontal lines.

From the gene expression analysis we found the KRT7 transcript increased by 19.8-fold in primary Ta tumours compared to normal bladder biopsies (Table 1). In order to evaluate the diagnostic value of KRT7 in bladder cancer, we looked at KRT7 expression in an expanded data set (evaluation data set 2) containing also muscle-invasive T2-4 tumours. Again, we found strong KRT7 transcript upregulation in Ta tumours (*N*=48), as well as in T2-4 tumours (*N*=24) compared to normal bladder biopsies (Figure 4). The median KRT7 expression in normal bladder biopsies was estimated to be 140.0±189.5, and in the cases of Ta and T2-4 tumours, 2775.1±1165.4 and 1192.7±698.7, respectively. This gives fold changes equal to 19.8-fold (*P*<0.05) and 8.5-fold (*P*<0.05), respectively.

**Figure 5:**
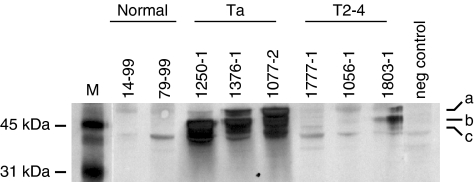
Expression of KRT7 protein in normal bladder biopsies, Ta, and T2-4 tumours analysed by Western blotting. Detection of three KRT7 protein isoforms; (a) isoform a=wild-type KRT7 (52 kDa), (b) isoform b (∼45 kDa), and (c) isoform c (∼42 kDa).

**Figure 6:**
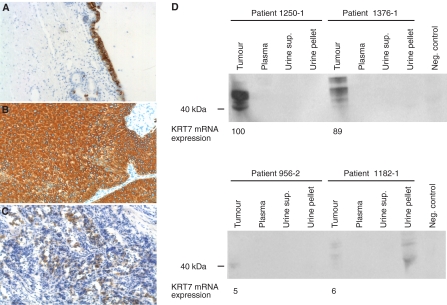
Immunohistochemical staining of KRT7 in (**A**) normal bladder, (**B**) Ta, and (**C**) T2-4 tumours. Original magnification: × 20. (**D**) Western blotting analysis of KRT7 protein expression in various biological samples from four individual bladder cancer patients. Protein extracts were obtained from tumour tissue, plasma, urine supernatant, and urine pellet. Extracted protein of leucocytes obtained from a control person was used as a negative control (neg. control). Expression of KRT7 transcript measured by microarray analysis shown in values relative to patient 1250-1.

By Western blotting analysis we found that the KRT7 protein level indeed was increased in Ta tumours (*N*=7) (Figure 5). In addition, the KRT7 protein level in most T2-4 tumours (*N*=6) was also increased, compared to normal biopsies (*N*=8). Surprisingly, several presumably cancer-specific KRT7 isoforms were observed. At least three major isoforms having different molecular weight were observed; isoform a (with MW equal to wild type KRT7=52 kDa), isoform b (∼45 kDa), and isoform c (∼42 kDa). We were able to detect the isoforms b and c in cancer tissue, but not in normal biopsies. We further extended the analysis to include KRT7 immunohistochemistry of normal bladder, Ta and T2-4 tumours (Figure 6A–C). In good agreement with previous reports (Jiang *et al*, 2001; Park *et al*, 2002), we found that KRT7 expression was highly epithelial cell specific, as the protein exclusively is expressed in either normal urothelial cells or cancer cells derived from urothelial cells.

To investigate whether KRT7 mRNA levels correlate with the protein levels, we simultaneously measured KRT7 gene expression (by microarrays) and protein expression (by Western blotting) of four Ta tumours. In all four cases, we found a correlation between KRT7 protein and mRNA expression (Figure 6D).

As, Ta tumours showed a high KRT7 expression we examined the potential of KRT7 as a new urine marker for early bladder cancer. We evaluated the KRT7 protein expression simultaneously in tumour tissue, plasma, urine supernatant, and urine pellet from four individuals diagnosed with Ta tumours (Figure 6D). By Western blotting analysis we were not able to detect KRT7 protein in neither plasma nor urine supernatant. As expected, we did not detect KRT7 protein in a preparation of leucocytes (negative control). However, we were able to detect KRT7 protein in urine pellets of some bladder cancer patients. The sensitivity of Western blotting was low, as only three out eight individuals were positive for KRT7 protein (not shown), but may be increased by using ELISA techniques.

## DISCUSSION

We have used DNA microarrays to study the gene expression in primary noninvasive bladder tumours of stage Ta. The purpose was to define classes of genes that alter in the early malignant transformation from normal bladder through primary Ta tumours of atypia grade PUNLMP, low-grade and high-grade. We identified a number of genes that show an altered expression in the early steps from normal bladder to Ta tumours. The latter characterised by a slight increase in number of cell layers, but otherwise very few cellular abnormalities, compared to normal cells. This early neoplastic process was accompanied by changes in expression of genes related to functional important classes, such as cytoskeleton (KRT7, KRT8, JUP, SDC1), protein folding (FABP6, GRSF1), transferase activity (CD24, LMNA), and transcription (HMGA1). The tissue composition between normal bladder wall and papillary tumours vary with respect to cellular content. However, previous studies have shown that the stromal cells are not very active in transcription leading to a low RNA level in those cells (Thykjaer *et al*, 2001), and the cold cup biopsies are superficial leading to an enrichment for urothelial cells. Further, our approach here is to define candidate genes that show a differential expression between normal mucosa and papillary tumours, and to confirm the level and location of the encoded protein, of some of those, by immunohistochemistry. In the part of the manuscript dealing with differences between various grades of atypia, the tissue composition is the same in the different groups.

In the present study we have identified several transcripts, which were previously shown to be overexpressed in cancer. Of particular interest is the SDC1 gene (Barbareschi *et al*, 2003; Mor *et al*, 2003), which in breast cancer is expressed at high levels, and is related to an aggressive phenotype, and poor clinical behavior (Barbareschi *et al*, 2003). The SDC1 transcript encodes a transmembrane heparan sulfate proteoglycan, which acts as a receptor of the extracellular matrix and thereby is involved in cell–cell adhesion, organisation of cell–matrix adhesion, and regulation of growth factor signalling. SDC1 is usually located to the cell membrane, and our finding of a cytoplasmic staning pattern in Ta tumours suggests a failure in intracellular trafficking, and could indicate loss of a functional SDC1 protein. This has in some tumours been related to a poor outcome due to reduced cell adhesion that promotes metastasis (Harada *et al*, 2003).

We identified a number of candidate informative genes that alter expression in high-grade tumours compared to PUNLMP/low-grade tumours. Of those, one of the most prominent was the CDK4 transcript. The CDK4 gene encodes a Ser/Thr protein kinase, which phophorylates retinoblastoma (Rb) and is an important cell cycle regulator (Ortega *et al*, 2002). In urinary bladder cancer, the chromosomal region housing CDK4 was found amplified, and chromosomal amplification has been documented to correlate with reduced survival and to be consistent with increased tumour grade, and genetic instability (Simon *et al*, 2002). In agreement with the gene expression profile, we found an increased protein expression of CDK4 in cancer cells from bladder tumours. We also found the gene expression of JUND altered in high-grade tumours. The protein encoded by the JUND gene is a member of the AP-1 family of transcription factors, which mediate the regulation of gene expression in response to extracellular signalling. The protein is a functional component of the AP-1 complex, and is strongly induced by UV-irradiation and may be involved in ML-1 cell apoptosis (Li *et al*, 2002). CDK4 and JUND are examples of genes, where both the gene expression and protein expression is consistently increased in tumours compared to normal urothelium.

Of the genes distinguishing Ta tumours from normal bladder biopsies, most were found upregulated. Similarly, we found most of the genes altered in high-grade tumours to be upregulated. This finding suggests that both neoplastic transformation of the urothelial cell layer, and also an increase in cellular atypia is connected with a massive transcription initiation event. Next, we searched for over-represented gene categories annotated within the biological process branch by the Gene Ontology Consortium (Ashburner *et al*, 2000). We found several over-represented gene categories within the list of differentially expressed genes in Ta tumours compared to normal bladder biopsies relating to both protein folding, mRNA splicing, energy pathways, glucose catabolism, and lipid metabolism. A neoplastic transformation of the urothelial cell-layer involves increasing energy turnover; therefore, increased glucose catabolism as well as lipid metabolism would be expected. Cells would also have an increased demand of mRNA splicing capacity. Among the genes distinguishing high-grade tumours from PUNLMP/low-grade tumours, we found over-represented gene categories related mainly to RNA processing and metabolism. Other categories were protein-nucleus import, and regulation of cell cycle. In agreement with this, cancer cells from high-grade tumours are distinguished from those of PUNLMP/low-grade tumours by abnormal cell size, shape, large hyperchromatic nuclei, and frequent mitoses.

When sorting the genes that were differentially expressed we used two approaches, one was very conservative (Benjamini–Hochberg correction for multiple comparisons) and was used for changes from normal bladder to Ta tumours. Another approach was used when looking at high-grade *vs* PUNLMP/low grades. This was due to the fact that the fold changes between the grade groups were lower, and a very conservative approach would remove all genes from the listing. We therefore used the *P*-value without correction for multiple comparisons, and named these ‘candidate informative’ genes. The generated list of genes seems biologically relevant (e.g. cell cycle, cell growth), and thus we believe that we gained information that is useful, instead of simply discarding the data. Of course, this means that such genes have to be proven by other methods and in other studies. In this case we found a separation into relevant subgroups using cluster analysis indicating robustness of the gene expression patterns within the groups.

The WHO/ISUP grading system (Epstein *et al*, 1998) is used to divide Ta tumours into one of four subcategories; papilloma, PUNLMP, low-grade, or high-grade. We performed an unsupervised clustering analysis and demonstrated that we could obtain grade-specific subgroups based on gene expression profiles. Two distinct clusters (PUNLMP subcluster/high-grade subcluster) were defined of both clinically and pathologically different tumours. We found a statistically significant differential distribution of PUNLMP and high-grade tumours to each of the two clusters. We compared the median time to recurrence of the two subcluster groups, and these were significantly different. The estimated median relapse intervals were only 7 months for high-grade subcluster tumours, and 63 months for PUNLMP subcluster tumours. Thus, it was possible to separate clinical distinct groups of Ta tumours on the basis of their gene expression pattern.

Cytokeratins belong to the intermediate filament family of cytoplasmic proteins and is normally expressed by epithelial cells. Cytoskeletal rearrangements seem to be an early change in the neoplastic process of the urothelium (Southgate *et al*, 1999; Mor *et al*, 2003; Sanchez-Carbayo *et al*, 2003). Consistent with the literature, we found members of the cytokeration family, especially KRT7, but also KRT8 highly expressed in Ta tumours (Southgate *et al*, 1999; Mor *et al*, 2003). Several previous studies have examined KRT7 expression in cancerous tissue of both primary and metastatic origin (Bassily *et al*, 2000; Tot, 2000; Park *et al*, 2002). Using comparison analysis, KRT7 was found as the most changed transcript with an increased expression of 19.8-fold in Ta, and 8.5-fold in T2-4 compared to normal bladder biopsies. Immunohistochemical stains for KRT7 showed cytoplasmic protein expression specific for cancer cells in both Ta and T2-4 tumours, but absent in stromal cells. Western blotting analysis using KRT7 specific antibody, consistently showed a high level of KRT7 protein in Ta tumours and most T2-4 tumours compared to normal bladder biopsies. Importantly, we did not find a KRT7 protein overexpression in individual cancer cells (which are derived from urothelial cells) compared to normal bladder cells. This suggests that the KRT7 expression profile could correlate to the number of cells expressing KRT7. Thus, the observed increase in KRT7 protein (and mRNA) could reflect cancer cell enrichment in Ta tumour biopsies, and might not only be a result of KRT7 overexpression. By Western blotting analysis, we found a correlation between KRT7 protein and mRNA expression. This was in agreement with what we showed previously for other keratins (Orntoft *et al*, 2002). We were not able to detect KRT7 protein in either plasma or urine from bladder cancer patients. In a few cases, we did detect KRT7 in urine pellet samples from patients, but with low sensitivity. Even though cancer cells in bladder tumours strongly expresses KRT7 protein, it cannot be used as a new urine marker for bladder cancer within this setup. However, an ELISA based method would be interesting to use with its higher sensitivity and better quantitative properties. Western blotting revealed the presence of at least three major isoforms of the KRT7 polypeptide. One isoform with a molecular weight similar to wild-type KRT7 (52 kDa), and two additional isoforms with molecular weights less than wild-type KRT7. As, it was only possible to detect isoform b and c in cancerous tissue, we speculate that these isoforms are cancer specific. Further studies are needed to reveal the origin of the two alternative KRT7 polypeptides isoforms, they may be the result of either post-translational modification or alternative splicing.

In conclusion, we have identified genes that alter expression in early phases of bladder tumour formation, and characterised the protein expression pattern of a number of those. We have identified genes contributing to the molecular signature of high-grade tumours, and shown, using clustering analysis, that primary Ta tumours can be subdivided according to grade of atypia. In addition, we have shown strong expression of both KRT7 transcript and protein in bladder cancer, the presence of KRT7 protein in urine pellets from bladder cancer patients, and the existence of three major isoforms of this molecule, mainly in malignant cells.

## Figures and Tables

**Table 1:** Differentially expressed genes in Ta tumours compared to normal bladder

**Gene transcript**	**Gene symbol^a^**	**Tentative gene function**	**Probeset ID**	***P*-value**	**FC^b^**
Keratin 7	**KRT7**	Cytoskeleton organisation	209016_s_at	7.51E-11^c^	19.8
IMP (inosine monophosphate) dehydrogenase 2	IMPDH2	RNA binding, spliceosome complex	201892_s_at	4.71E-09^c^	2.8
OGT(O-Glc-NAc transferase)-interacting protein 106 kDa	OIP106	DNA binding, G -protein coupled receptor protein signalling pathway	214924_s_at	7.45E-09^c^	3.9
Junction plakoglobin	JUP	Cytoskeleton, cell adhesion	201015_s_at	8.31E-09^c^	2.8
Keratin 8	KRT8	Cytoskeleton, protein-nucleus import	209008_x_at	9.21E-09^c^	5.4
Protein tyrosine phosphatase, receptor type, F	PTPRF	Transmembrane receptor protein tyrosine phosphatase signalling pathway	200635_s_at	1.07E-08^c^	2.9
Syndecan 1	**SDC1**	Cytoskeletal protein binding, integral to plasma membrane	201286_at	1.51E-08^c^	3.8
High mobility group AT-hook 1	HMGA1	Transcription factor	206074_s_at	3.48E-08^c^	2.2
Protein phosphatase1, regulatory (inhibitor) subunit 14B	PPP1R14B	Phosphoprotein inhibitor	212680_x_at	4.98E-08^c^	2.5
Agrin	AGRN	Function unknown	212285_s_at	5.18E-08^c^	2.1
Fatty acid binding protein 6, ileal (gastrotropin)	FABP6	Heat shock protein activity, protein folding	210445_at	6.90E-08^c^	3.5
Slingshot 3	SSH-3	Acid phosphatase activity	219241_x_at	7.99E-08^c^	2.0
EST		Function unknown	216379_x_at	9.99E-08^c^	3.1
Syndecan 1	**SDC1**	Cytoskeletal protein binding, integral to plasma membrane	201287_s_at	1.16E-07^c^	4.3
Hypoxia-inducible factor prolyl 4-hydroxylase	PH-4	Oxidoreductase activity	222125_s_at	1.33E-07^c^	2.0
G-rich RNA sequence binding factor 1	GRSF1	Isomerase activity, protein folding	201520_s_at	1.51E-07^c^	3.6
CD24 antigen (small-cell lung carcinoma cluster 4 antigen)	CD24	Cytoplasm, transferase activity	209771_x_at	1.67E-07^c^	3.1
Lamin A/C	LMNA	Protein kinase C activation, transferase activity	203411_s_at	1.82E-07^c^	2.2
FAT tumour suppressor homolog 1 (Drosophila)	FAT	Negative regulation of cell cycle	201579_at	1.94E-07^c^	3.2
Aminoacylase 1	ACY1	Metallopeptidase activity	202740_at	2.11E-07^c^	2.4

a Protein localisation measured by IHC shown in bold.

b Fold change (median Ta/median *N*).

c Adjusted for multiplicity *P*<0.05.

**Table 2:** Informative genes that distinguish high grade Ta tumours from PUNLMP/low grade Ta tumours

**Gene transcript**	**Gene symbol^a^**	**Tentative gene function**	**Probeset ID**	***P*-value**	**FC^b^**
Hypothetical protein FLJ13725	FLJ13725	Function unknown	45749_at	3.87E-05^c^	0.7
Cyclin-dependent kinase 4	**CDK4**	Regulation of cell cycle	202246_s_at	4.56E-05^c^	1.4
Ras homologue gene family, member Q	ARHQ	GTP binding, Rho small monomeric GTPase activity	212119_at	9.17E-05^c^	1.7
Diazepam binding inhibitor (GABA receptor modulator, acyl-Coenzyme A binding protein)	DBI	Benzodiazepine receptor binding	202428_x_at	1.20E-04^c^	1.9
Tyrosine 3-monooxygenase/tryptophan 5-monooxygenase activation protein, zeta polypeptide	YWHAZ	Protein domain specific binding	200640_at	1.39E-04^c^	1.6
H2A histone family, member Z	H2AFZ	DNA binding, nucleosome assembly, nucleus	213911_s_at	2.02E-04^c^	2.4
Diazepam binding inhibitor (GABA receptor modulator, acyl-coenzyme A binding protein)	DBI	Benzodiazepine receptor binding	211070_x_at	2.42E-04^c^	2.0
Voltage-dependent anion channel 3	VDAC3	Voltage-dependent anion channel porin activity	208845_at	2.46E-04^c^	1.4
Hypothetical protein MAC30	MAC30	Regulation of cell growth	212282_at	4.50E-04^c^	3.1
Interleukin enhancer binding factor 2, 45 kDa	ILF2	RNA polymerase II transcription factor activity, nucleus	200052_s_at	4.51E-04^c^	1.8
Likely ortholog of mouse immediate early response, erythropoietin 4	LEREPO4	Nucleic acid binding	201593_s_at	5.11E-04^c^	1.7
Hypothetical protein MAC30	MAC30	Regulation of cell growth	212281_s_at	5.92E-04^c^	3.6
Proteasome (prosome, macropain) subunit, beta type, 7	PSMB7	Endopeptidase activity, ubiquitin-dependent protein catabolism	200786_at	6.85E-04^c^	1.7
Transgelin 2	TAGLN2	Muscle development	200916_at	7.64E-04^c^	1.6
Proteasome (prosome, macropain) subunit, alpha type, 6	PSMA6	RNA binding, endopeptidase activity, ubiquitin-dependent protein catabolism	208805_at	8.10E-04^c^	1.4
Aldo-keto reductase family 1, member A1 (aldehyde reductase)	AKR1A1	Oxidoreductase activity	201900_s_at	8.22E-04^c^	1.3
Peroxiredoxin 1	PRDX1	Cell proliferation, peroxidase activity	208680_at	8.23E-04^c^	1.5
Chromosome 13 open reading frame 12	C13orf12	Function unknown	217769_s_at	8.42E-04^c^	1.4
Jun D proto-oncogene	**JUND**	Regulation of transcription from Pol II promoter, transcription factor activity	203752_s_at	8.57E-04^c^	1.8
Formin binding protein 3	FNBP3	mRNA splicing, signal transduction	213729_at	8.78E-04^c^	1.9

a Protein localisation measured by IHC shown in bold.

b Fold change (median high grade/median PUNLMP/low grade).

c Adjusted for multiplicity *P*>0.05.

**Table 3:** Identification of over-represented gene categories

**Gene category^a^**	**EASE score**
*Analysis of 230 differentially expressed genes in Ta tumours compared to normal bladder*	
Protein folding	3.28E-04
Alcohol metabolism	2.44E-03
mRNA splicing	2.58E-03
Energy derivation by oxidation of organic compounds	3.75E-03
Energy pathways	4.19E-03
RNA splicing	6.15E-03
Glucose catabolism	8.59E-03
Obsolete biological process	8.66E-03
Main pathways of carbohydrate metabolism	8.71E-03
Lipid metabolism	8.73E-03
Hexose catabolism	1.32E-02
Alcohol catabolism	1.32E-02
Monosaccharide catabolism	1.32E-02
Catecholamine metabolism	1.62E-02
	
*Analysis of 86 candidate informative genes expressed in high-grade Ta tumours compared to PUNLMP/low-grade tumours*	
RNA processing	1.46E-03
RNA metabolism	2.29E-03
RNA modification	3.54E-03
Protein-nucleus importdocking	4.60E-03
Nucleoside monophosphate metabolism	5.16E-03
Nucleoside monophosphate biosynthesis	5.16E-03
Protein-nucleus import	5.89E-03
DNA metabolism	1.22E-02
Nucleocytoplasmic transport	2.07E-02
Regulation of cell cycle	2.79E-02

a Annotated within GO biological process.

## Supplementary Material

Supplementary Table 1

Supplementary Table 1 Continued-1

Supplementary Table 1 Continued-2

Supplementary Table 1 Continued-3

Supplementary Table 1 Continued-4

Supplementary Table 1 Continued-5

Supplementary Table 1 Continued-6

Supplementary Table 1 Continued-7

Supplementary Table 2

Supplementary Table 2 Continued-1

Supplementary Table 2 Continued-2
