# Supplementary material for: Gene expression profiling of noninvasive primary urothelial tumours using microarrays
Source: Br J Cancer. 2005 Nov 1;93(10):1182–90. doi: 10.1038/sj.bjc.6602813 (PMC2361501; doi:10.1038/sj.bjc.6602813)
Supplement: Supplementary Table 1 [file 93-6602813x1.pdf]

Supplementary table 1. Differentially expressed genes in Ta tumors compared to normal bladder.

Gene transcript	Gene symbol	Unigene	Probeset ID	p-value	FC±	Adjusted p<0.05
keratin 7	KRT7	Hs.356123	209016_s_at	7.51E-11	19.8	yes
IMP (inosine monophosphate) dehydrogenase 2	IMPDH2	Hs.173255	201892_s_at	4.71E-09	2.8	yes
OGT(O-Glc-NAc transferase)-interacting protein 106 KDa	OIP106	Hs.279518	214924_s_at	7.45E-09	3.9	yes
junction plakoglobin	JUP	Hs.179526	201015_s_at	8.31E-09	2.8	yes
keratin 8	KRT8	Hs.439683	209008_x_at	9.21E-09	5.4	yes
protein tyrosine phosphatase, receptor type, F	PTPRF	Hs.75216	200635_s_at	1.07E-08	2.9	yes
syndecan 1	SDC1	Hs.221589	201286_at	1.51E-08	3.8	yes
high mobility group AT -hook 1	HMGA1	Hs.2785	206074_s_at	3.48E-08	2.2	yes
protein phosphatase 1, regulatory (inhibitor) subunit 14B	PPP1R14B	Hs.14376	212680_x_at	4.98E-08	2.5	yes
agrin	AGRN	Hs.2785	212285_s_at	5.18E-08	2.1	yes
fatty acid binding protein 6, ileal (gastrotropin)	FABP6	Hs.180414	210445_at	6.90E-08	3.5	yes
slingshot 3	SSH-3	Hs.15871	219241_x_at	7.99E-08	2.0	yes
EST		Hs.172550	216379_x_at	9.99E-08	3.1	yes
syndecan 1	SDC1	Hs.82109	201287_s_at	1.16E-07	4.3	yes
hypoxia-inducible factor prolyl 4 -hydroxylase	PH-4	Hs.271224	222125_s_at	1.33E-07	2.0	yes
G-rich RNA sequence binding factor 1	GRSF1	Hs.381072	201520_s_at	1.51E-07	3.6	yes
CD24 antigen (small cell lung carcinoma cluster 4 antigen)	CD24	Hs.411855	209771_x_at	1.67E-07	3.1	yes
lamin A/C	LMNA	Hs.172690	203411_s_at	1.82E-07	2.2	yes
FAT tumor suppressor homolog 1 (Drosophila)	FAT	Hs.118638	201579_at	1.94E-07	3.2	yes
aminoacylase 1	ACY1	Hs.76719	202740_at	2.11E-07	2.4	yes
nucleostemin	NS	Hs.390667	217850_at	2.18E-07	3.3	yes
CD24 antigen (small cell lung carcinoma cluster 4 antigen)	CD24	Hs.375108	208651_x_at	2.41E-07	2.6	yes
polypyrimidine tract binding protein 1	PTBP1	Hs.14376	212015_x_at	2.44E-07	2.2	yes
solute carrier family 38, member 1	SLC38A1	Hs.432752	218237_s_at	2.65E-07	4.2	yes
solute carrier family 25 (mitochondrial carrier; adenine nucleotide translocator), member 4	SLC25A4	Hs.105894	202826_at	2.84E-07	3.1	yes
heterogeneous nuclear ribonucleoprotein U (scaffold attachment factor A)	HNRPU	Hs.166463	200593_s_at	2.88E-07	2.7	yes
integrin, beta 4	ITGB4	Hs.377028	204990_s_at	2.99E-07	2.9	yes
KIAA0303 protein	KIAA0303		210958_s_at	3.59E-07	1.5	yes
TBC1 domain family, member 3	TBC1D3	Hs.432574	209403_at	3.62E-07	2.0	yes
yeast Sec31p homolog	KIAA0905	Hs.411855	210616_s_at	3.62E-07	1.9	yes
serine/threonine kinase 24 (STE20 homolog, yeast)	STK24	Hs.155560	208854_s_at	3.85E-07	3.3	yes
phosphofructokinase, liver	PFKL	Hs.408507	201102_s_at	4.86E-07	1.8	yes
protein tyrosine phosphatase, receptor type, F	PTPRF	Hs.75216	200636_s_at	4.91E-07	3.0	yes
diacylglycerol kinase, alpha 80kDa	DGKA	Hs.18141	203385_at	5.04E-07	2.2	yes
RAP1, GTPase activating protein 1	RAP1GA1	Hs.241567	203911_at	5.41E-07	1.6	yes
