# Supplementary material for: Gene expression profiling of noninvasive primary urothelial tumours using microarrays
Source: Br J Cancer. 2005 Nov 1;93(10):1182–90. doi: 10.1038/sj.bjc.6602813 (PMC2361501; doi:10.1038/sj.bjc.6602813)
Supplement: Supplementary Table 1 Continued-1 [file 93-6602813x2.pdf]

Supplementary table 1. Continued-1.

Gene transcript	Gene symbol	Unigene	Probeset ID	p-value	FC±	Adjusted p<0.05
ladinin 1	LAD1	Hs.511936	203287_at	6.34E-07	1.9	yes
integrin beta 4 binding protein	ITGB4BP	Hs.374638	210213_s_at	6.77E-07	2.7	yes
N-acylaminoacyl-peptide hydrolase	APEH	Hs.81361	201283_s_at	7.30E-07	3.3	yes
solute carrier family 20 (phosphate transporter), member 1	SLC20A1	Hs.75432	201920_at	7.40E-07	4.3	yes
keratin 19	KRT19	Hs.406013	201650_at	7.95E-07	5.4	yes
vascular endothelial growth factor	VEGF	Hs.355861	210512_s_at	8.56E-07	4.7	yes
elongation of very long chain fatty acids (FEN1/Elo2, SUR4/Elo3, yeast) -like 1	ELOVL1	Hs.79625	218028_at	8.85E-07	2.3	yes
calcium/calmodulin -dependent protein kinase II	CaMKIIN alpha	Hs.132246	218309_at	9.72E-07	4.1	yes
agrin	AGRN	Hs.16003	217419_x_at	1.03E-06	1.9	yes
beta 5-tubulin	OK/SW - cl.56	Hs.73793	211714_x_at	1.07E-06	2.5	yes
chromosome 20 open reading frame 149	C20orf149	Hs.388130	218010_x_at	1.07E-06	2.0	yes
catenin (cadherin-associated protein), delta 1	CTNND1	Hs.168913	208862_s_at	1.09E-06	1.9	yes
beta 5-tubulin	OK/SW - cl.56	Hs.23881	209026_x_at	1.11E-06	2.4	yes
heterogeneous nuclear ribonucleoprotein A1	HNRPA1	Hs.374638	214280_x_at	1.13E-06	2.3	yes
filamin B, beta (actin binding protein 278)	FLNB	Hs.308709	208613_s_at	1.16E-06	2.0	yes
adipose specific 2	APM2	Hs.436441	203571_s_at	1.25E-06	4.0	yes
serologically defined breast cancer antigen 84	SDBCAG 84	Hs.142	216032_s_at	1.25E-06	2.4	yes
slingshot 3	SSH-3	Hs.29173	51192_at	1.37E-06	2.0	yes
GPI-anchored metastasisassociated protein homolog	C4.4A	Hs.59889	204952_at	1.39E-06	2.6	yes
cyclin L2	CCNL2	Hs.143601	221427_s_at	1.40E-06	2.4	yes
S-adenosylhomocysteine hydrolase	AHCY	Hs.183994	200903_s_at	1.59E-06	3.1	yes
aldolase A, fructose-bisphosphate	ALDOA	Hs.406693	200966_x_at	1.67E-06	2.0	yes
NADH:ubiquinone oxidoreductase MLRQ subunit homolog	LOC56901	Hs.433615	214096_s_at	1.70E-06	1.8	yes
WD repeat domain 6	WDR6	Hs.381079	217734_s_at	1.73E-06	1.7	yes
leucine rich repeat (in FLII) interacting protein 1	LRRFIP1	Hs.180577	211452_x_at	1.73E-06	3.3	yes
protein tyrosine phosphatase, receptor type, F	PTPRF	Hs.75216	200637_s_at	1.77E-06	5.5	yes
similar to Nu clear envelope pore membrane protein POM 121 (Pore membrane protein of 121 kDa) (P145)	na	Hs.440900	213360_s_at	1.77E-06	1.8	yes
mannosyl (alpha-1,3-)-glycoprotein beta-1,2-N-acetylglucosaminyltransferase	MGAT1	Hs.368741	201126_s_at	2.04E-06	2.0	yes
ribonuclease 6 precursor	RNASE6PL	Hs.313544	217983_s_at	2.07E-06	3.5	yes
tripartite motif -containing 28	TRIM28	Hs.434937	200990_at	2.09E-06	2.5	yes
calpain 1, (mu/l) large subunit	CAPN1	Hs.374638	200752_s_at	2.18E-06	1.9	yes
OGT(O-Glc-NAc transferase)-interacting protein 106 KDa	OIP106	Hs.7768	202080_s_at	2.46E-06	1.8	yes
involucrin	IVL	Hs.311765	214599_at	2.46E-06	2.4	yes
glucose regulated protein, 58kDa	GRP58	Hs.387667	208612_at	2.51E-06	3.4	yes
pogo transposable element with KRAB domain	POGK	Hs.25597	218229_s_at	3.00E-06	2.4	yes
fatty acid synthase	FASN	Hs.73793	212218_s_at	3.13E-06	2.5	yes
CD24 antigen (small cell lung carcinoma cluster 4 antigen)	CD24	Hs.375108	266_s_at	3.13E-06	3.2	yes
