# Supplementary material for: Gene expression profiling of noninvasive primary urothelial tumours using microarrays
Source: Br J Cancer. 2005 Nov 1;93(10):1182–90. doi: 10.1038/sj.bjc.6602813 (PMC2361501; doi:10.1038/sj.bjc.6602813)
Supplement: Supplementary Table 1 Continued-2 [file 93-6602813x3.pdf]

Supplementary table 1. Continued-2.

Gene transcript	Gene symbol	Unigene	Probeset ID	p-value	FC±	Adjusted p<0.05
heat shock 70kDa protein 9B (mortalin -2)	HSPA9B	Hs.410578	200692_s_at	3.24E-06	2.1	yes
small nuclear ribonucleoprotein polypeptides B and B1	SNRPB		213175_s_at	3.43E-06	2.0	yes
HLA -B associated transcript 1	BAT1	Hs.254042	200041_s_at	3.52E-06	2.7	yes
heme-regulated initiation factor 2 -alpha kinase	HRI	Hs.8737	217736_s_at	3.55E-06	2.4	yes
fer-1-like 4 (C. elegans)	FER1L4	Hs.72222	222245_s_at	3.58E-06	2.0	yes
general transcription factor II, i	GTF2I	Hs.2340	201065_s_at	3.61E-06	3.3	yes
likely ortholog of mouse exocyst component protein 70 kDa homolog (S. cerevisiae) Exo70: exocyst component protein 70 kDa homolog (S. cerevisiae)	EXO70	Hs.172550	212034_s_at	3.98E-06	2.1	yes
transgelin 2	TAGLN2	Hs.388004	200916_at	3.99E-06	1.7	yes
fuse-binding protein -interacting repressor	SIAHBP1	Hs.241567	209899_s_at	4.00E-06	1.9	yes
amyloid beta (A4) precursor -like protein 2	APLP2	Hs.406532	208702_x_at	4.08E-06	2.3	yes
cartilage acidic protein 1	CRTAC1	Hs.326444	221204_s_at	4.24E-06	7.3	yes
KDEL (Lys -Asp-Glu-Leu) endoplasmic reticulum protein retention receptor 1	KDELR1	Hs.406504	200922_at	4.29E-06	1.9	yes
ribonuclease 6 precursor	RNASE6PL	Hs.388130	217984_at	4.39E-06	2.3	yes
CD24 antigen (small cell lung carcinoma cluster 4 antigen)	CD24	Hs.75860	208650_s_at	4.52E-06	5.6	yes
vascular endothelial growth factor	VEGF	Hs.170333	212171_x_at	4.53E-06	2.8	yes
TH1-like (Drosophila)	TH1L	Hs.5184	220607_x_at	4.57E-06	3.2	yes
major histocompatibility complex, class II, DP beta 1	HLA -DPB1	Hs.387567	201137_s_at	4.79E-06	0.4	yes*
fibroblast growth factor receptor 3 (achondroplasia, thanatophoric dwarfism)	FGFR3	Hs.433797	204379_s_at	4.94E-06	5.3	yes
ladinin 1	LAD1	Hs.212102	216641_s_at	4.95E-06	2.0	yes
solute carrier family 6 (neurotransmitter transporter, creatine), member 8	SLC6A8	Hs.457063	202219_at	5.19E-06	3.4	yes
fatty acid bin ding protein 5 (psoriasis-associated)	FABP5	Hs.444947	202345_s_at	5.28E-06	5.8	yes
eukaryotic translation initiation factor 3, subunit 8, 110kDa	EIF3S8	Hs.436549	210949_s_at	5.3E-06	2.6	yes
RNA binding motif, single stranded interacting protein 1	RBMS1	Hs.375108	209868_s_at	5.84E-06	1.9	yes
FXYD domain containing ion transport regulator 3	FXYD3	Hs.202453	202488_s_at	6.08E-06	1.8	yes
keratin 17	KRT17	Hs.83190	212236_x_at	6.19E-06	3.6	yes
T-box 3 (ulnar mammary syndrome)	TBX3	Hs.371804	219682_s_at	6.38E-06	2.6	yes
MCM7 minichromosome maintenance deficient 7 (S. cerevisiae)	MCM7	Hs.4112	208795_s_at	6.6E-06	1.8	yes
voltage-dependent anion channel 1	VDAC1	Hs.325530	212038_s_at	6.76E-06	2.8	yes
ret finger protein	RFP	Hs.436441	212116_at	6.83E-06	1.6	yes
Homo sapiens, clone MGC:8772 IMAGE:3862861, mRNA, complete cds		Hs.432850	213166_x_at	6.86E-06	2.2	yes
ubiquitin -activating enzyme E1 (A1S9T and BN75 temperature sensitivity complementing)	UBE1	Hs.107720	200964_at	7.16E-06	1.9	yes
polypyrimidine tract bin ding protein 1	PTBP1	Hs.172550	212016_s_at	7.41E-06	1.9	yes
SREBP CLEAVAGE -ACTIVATING PROTEIN	SCAP	Hs.356729	212329_at	7.72E-06	2.0	yes
FK506 binding protein 1A, 12kDa	FKBP1A	Hs.173902	200709_at	8E-06	1.9	yes
hypothetical protein FLJ21918	FLJ21918	Hs.12727	219395_at	8.02E-06	2.1	yes
oxidase (cytochrome c) assembly 1-like	OXA1L	Hs.7744	208717_at	8.09E-06	1.4	yes
