# Supplementary material for: Gene expression profiling of noninvasive primary urothelial tumours using microarrays
Source: Br J Cancer. 2005 Nov 1;93(10):1182–90. doi: 10.1038/sj.bjc.6602813 (PMC2361501; doi:10.1038/sj.bjc.6602813)
Supplement: Supplementary Table 1 Continued-4 [file 93-6602813x5.pdf]

Supplementary table 1. Continued-4.

Gene transcript	Gene symbol	Unigene	Probeset ID	p-value	FC±	Adjusted p<0.05
hypothetical protein FLJ20618	FLJ20618	Hs.52184	222244_s_at	8.23E-06	2.2	yes
splicing factor 1	SF1	Hs.406458	208313_s_at	8.28E-06	1.6	yes
actin, gamma 1	ACTG1	Hs.446579	211995_x_at	8.52E-06	2.3	yes
NSFL1 (p97) cofactor (p47)	NSFL1C	Hs.12865	220248_x_at	8.59E-06	2.1	yes
		Hs.429975	217256_x_at	8.62E-06	1.5	yes
NAD(P)H dehydrogenase, quinone 1	NQO1	Hs.73793	210519_s_at	8.85E-06	3.7	yes
non-metastatic cells 2, protein (NM23B) expressed in	NME2	Hs.169902	201268_at	8.87E-06	2.6	yes
LSM4 homolog, U6 small nuclear RNA associated (S. cerevisiae)	LSM4	Hs.301350	202736_s_at	9.22E-06	2.7	yes
low density lipoprotein receptor -related protein associated protein 1	LRPAP1	Hs.368409	201186_at	9.59E-06	1.8	yes
granulin	GRN	Hs.172550	211284_s_at	9.73E-06	1.7	yes
actin, gamma 1	ACTG1	Hs.437096	212363_x_at	9.75E-06	2.2	yes
adaptor-related protein complex 2, mu 1 subunit	AP2M1	Hs.433892	200613_at	9.75E-06	2.6	yes
thioredoxin interacting protein	TXNIP	Hs.433612	201009_s_at	1.00E-05	2.3	yes
lamin A/C	LMNA	Hs.404814	212086_x_at	1.04E-05	1.7	yes
proteasome (prosome, macropain) subunit, beta type, 3	PSMB3	Hs.73527	201400_at	1.05E-05	2.2	yes
catenin (cadherin-associated protein), delta 1	CTNND1	Hs.143323	211240_x_at	1.06E-05	1.6	yes
v-myc myelocytomatosis viral oncogene homolog (avian)	MYC	Hs.408061	202431_s_at	1.06E-05	2.4	yes
RNA binding motif, single stranded interacting protein 1	RBMS1	Hs.57301	207266_x_at	1.11E-05	1.8	yes
heterogeneous nuclear ribonucleoprotein A/B	HNRPAB	Hs.433416	201277_s_at	1.12E-05	2.1	yes
pM5 protein	PM5		217225_x_at	1.13E-05	1.7	yes
Putative prostate cancer tumor suppressor	N33	Hs.170160	209228_x_at	1.16E-05	2.6	yes
RAB2, member RAS oncogene family-like	RAB2L	Hs.82045	209110_s_at	1.24E-05	1.9	yes
amyloid beta (A4) precursor -like protein 2	APLP2	Hs.279518	208703_s_at	1.26E-05	2.7	yes
amyloid beta (A4) precursor -like protein 2	APLP2	Hs.183706	214875_x_at	1.30E-05	2.4	yes
fusion, deriv ed from t(12;16) malignant liposarcoma	FUS	Hs.178551	200959_at	1.31E-05	1.7	yes
		Hs.457063	215299_x_at	1.32E-05	2.6	yes
protein phosphatase 2 (formerly 2A), regulatory subunit A (PR 65), alpha isoform	PPP2R1A	Hs.184233	200695_at	1.35E-05	1.5	yes
aldolase A, fructose-bisphosphate	ALDOA	Hs.157091	214687_x_at	1.36E-05	1.9	yes
peptidylprolyl isomerase F (cyclophilin F)	PPIF	Hs.82793	201489_at	1.36E-05	2.2	yes
lysophosphatidic acid phosphatase	ACP6	Hs.100071	218795_at	1.36E-05	2.7	yes
ring finger protein 44	RNF44	Hs.244318	203286_at	1.44E-05	2.1	yes
t-complex 1	TCP1	Hs.151134	208778_s_at	1.45E-05	2.5	yes
transgelin 2	TAGLN2	Hs.212787	210978_s_at	1.47E-05	2.0	yes
hypothetical protein LOC162427	LOC162427	Hs.120197	212697_at	1.58E-05	2.3	yes
solute carrier family 2 (facilitated glucose transporter), member 1	SLC2A1	Hs.433455	201250_s_at	1.58E-05	1.8	yes
F11 receptor	F11R	Hs.414880	221664_s_at	1.58E-05	2.6	yes
RNA binding motif, single stranded interacting protein 1	RBMS1	Hs.511763	203748_x_at	1.63E-05	2.0	yes
keratin 17	KRT17	Hs.152096	205157_s_at	1.69E-05	4.9	yes
			216640_s_at	1.73E-05	2.6	yes
eukaryotic translation initiation factor 3,	EIF3S8	Hs.192425	200647_x_at	1.73E-05	1.9	yes
