# Supplementary material for: Gene expression profiling of noninvasive primary urothelial tumours using microarrays
Source: Br J Cancer. 2005 Nov 1;93(10):1182–90. doi: 10.1038/sj.bjc.6602813 (PMC2361501; doi:10.1038/sj.bjc.6602813)
Supplement: Supplementary Table 1 Continued-5 [file 93-6602813x6.pdf]

Supplementary table 1. Continued-5.

Gene transcript	Gene symbol	Unigene	Probeset ID	p-value	FC$^{\pm}$	Adjusted p<0.05
N-myc downstream regulated gene 1	NDRG1	Hs.318567	200632_s_at	1.76E-05	2.5	yes
hypothetical protein LOC283820	LOC283820	Hs.227823	221853_s_at	1.86E-05	2.3	yes
hypothetical protein MGC5178	MGC5178	Hs.114309	209607_x_at	1.94E-05	1.8	yes
3-hydroxy-3-methylglutaryl -Coenzyme A synthase 2 (mitochondrial)	HMG CS2	Hs.1420	204607_at	1.97E-05	6.2	yes
tubulin, beta, 2	TUBB2	Hs.426324	213726_x_at	2.03E-05	2.1	yes
CpG binding protein	CGBP	Hs.180933	48580_at	2.04E-05	1.5	yes
retinoic acid induced 17	RAI17	Hs.440382	212124_at	2.09E-05	2.1	yes
phosphoprotein regulated by mitogenic pathways	C8FW	Hs.388375	202241_at	2.1E-05	2.3	yes
basigin (OK blood group)	BSG	Hs.301882	208677_s_at	2.1E-05	2.4	yes
adducin 1 (alpha)	ADD1	Hs.271511	214736_s_at	2.11E-05	1.7	yes
glutathione S-transferase subunit 13 homolog	LOC51064	Hs.434986	217751_at	2.12E-05	2.3	yes
vascular endothelial growth factor	VEGF	Hs.127951	211527_x_at	2.25E-05	2.8	yes
tyrosine 3-monooxygenase/tryptophan 5-monooxygenase activation protein, epsilon polypeptide	YWHAE	Hs.406504	210996_s_at	2.27E-05	2.3	yes
Putative prostate cancer tumor suppressor	N33	Hs.168357	213423_x_at	2.31E-05	2.6	yes
sulfotransferase family, cytosolic, 1A, phenol-preferring, member 3	SULT1A3	Hs.406515	210580_x_at	2.32E-05	2.6	yes
KIAA0100 gene product	KIAA0100	Hs.309517	201729_s_at	2.32E-05	2.4	yes
peptidylprolyl isomerase F (cyclophilin F)	PPIF	Hs.381072	201490_s_at	2.38E-05	2.6	yes
FK506 binding protein 1A, 12kDa	FKBP1A	Hs.188401	210186_s_at	2.44E-05	1.9	yes
glucose phosphate isomerase	GPI	Hs.406701	208308_s_at	2.46E-05	2.8	yes
hydroxyacyl-Coenzyme A dehydrogenase/3-ketoacyl-Coenzyme A thiolase/enoyl-Coenzyme A hydratase (trifunctional protein), alpha subunit	HADHA	Hs.476435	208631_s_at	2.5E-05	1.9	yes
peroxisome proliferative activated receptor, gamma	PPARG	Hs.440835	208510_s_at	2.51E-05	3.3	yes
karyopherin (importin) beta 1	KPNB1	Hs.422340	208974_x_at	2.52E-05	2.9	yes
hypothetical protein FLJ20700	FLJ20700	Hs.241567	207730_x_at	2.62E-05	2.0	yes
apolipoprotein L, 1	APOL1	Hs.170222	209546_s_at	2.63E-05	2.6	yes
elongation of very long chain fatty acids (FEN1/Elo2, SUR4/Elo3, yeast) -like 1	ELOVL1	Hs.25597	57163_at	2.66E-05	2.2	yes
heat shock 70kDa protein 8	HSPA8	Hs.5215	210338_s_at	2.66E-05	4.0	yes
solute carrier family 9 (sodium/hydrogen exchanger), isoform 1 (antiporter, Na+/H+ , amiloride sensitive)	SLC9A1	Hs.454713	209453_at	2.73E-05	1.4	yes
zinc finger protein 36, C3H type -like 2	ZFP36L2	Hs.82109	201369_s_at	2.74E-05	2.6	yes
procollagen-proline, 2-oxoglutarate 4-dioxygenase (proline 4-hydroxylase), beta polypeptide (protein disulfide isomerase; thyroid hormone binding protein p55)	P4HB	Hs.388163	200656_s_at	2.87E-05	2.9	yes
ribosomal protein L8	RPL8	Hs.78040	200936_at	2.95E-05	2.1	yes
non-metastatic cells 1, protein (NM23A) expressed in	NME1	Hs.14376	201577_at	2.98E-05	2.4	yes
CD24 antigen (small cell lung carcinoma cluster 4 antigen)	CD24	Hs.375108	209772_s_at	2.99E-05	1.8	yes
FK506 binding protein 1A, 12kDa	FKBP1A	Hs.221447	214119_s_at	3.06E-05	1.5	yes
calnexin	CANX	Hs.240013	208852_s_at	3.09E-05	2.6	yes
SWI/SNF related, matrix associated, actin dependent regulator of chromatin, subfamily f, member 1	SMARCF1	Hs.438767	212152_x_at	3.12E-05	1.4	yes
