# Supplementary material for: Gene expression profiling of noninvasive primary urothelial tumours using microarrays
Source: Br J Cancer. 2005 Nov 1;93(10):1182–90. doi: 10.1038/sj.bjc.6602813 (PMC2361501; doi:10.1038/sj.bjc.6602813)
Supplement: Supplementary Table 1 Continued-6 [file 93-6602813x7.pdf]

Supplementary table 1. Continued-6.

Gene transcript	Gene symbol	Unigene	Probeset ID	p-value	FC[±]	Adjusted p<0.05
v-erb-b2 erythroblastic leukemia viral oncogene homolog 2, neuro/glioblastoma derived oncogene homolog (avian)	ERBB2	Hs.18141	216836_s_at	3.12E-05	2.2	yes
dolichyl -diphosphooligosaccharide-protein glycosyltransferase	DDOST	Hs.375108	208675_s_at	3.17E-05	2.5	yes
non-POU domain containing, octamer-binding	NONO	Hs.147391	210470_x_at	3.21E-05	1.8	yes
putative DNA/chromatin binding motif	PLU-1	Hs.79474	211202_s_at	3.22E-05	1.7	yes
HLA -B associated transcript 3	BAT3	Hs.83753	213318_s_at	3.29E-05	2.6	yes
casein kinase 2, beta polypeptide	CSNK2B	Hs.78909	201390_s_at	3.31E-05	1.7	yes
beta 5-tubulin	OK/SW-cl.56	Hs.273330	212320_at	3.34E-05	1.6	yes
annexin A10	ANXA10	Hs.74562	210143_at	3.34E-05	6.0	yes
hypothetical protein FLJ20154	FLJ20154	Hs.334707	202809_s_at	3.37E-05	1.4	yes
tyrosine 3-monooxygenase/tryptophan 5-monooxygenase activation protein, zeta polypeptide	YWHAZ	Hs.386834	200640_at	3.39E-05	3.1	yes
protein phosphatase 1, catalytic subunit, alpha isoform	PPP1CA	Hs.356181	200846_s_at	3.45E-05	2.2	yes
filamin B, beta (actin binding protein 278)	FLNB	Hs.476435	208614_s_at	3.46E-05	1.9	yes
KRAB zinc finger protein KR18	KR18	Hs.273415	214715_x_at	3.54E-05	2.0	yes
granulin	GRN	Hs.169992	216041_x_at	3.56E-05	1.9	yes
heat shock 90kDa protein 1, alpha	HSPCA	Hs.156316	211968_s_at	3.59E-05	3.9	yes
death-associated protein kinase 1	DAPK1	Hs.233950	203139_at	3.64E-05	2.0	yes
polypyrimidine tract binding protein 1	PTBP1	Hs.166011	211271_x_at	3.67E-05	2.0	yes
6-phosphogluconolactonase	PGLS	Hs.197922	218388_at	3.69E-05	2.1	yes
Homo sapiens Alu repeat (LNX1) mRNA sequence		Hs.180577	216187_x_at	3.7E-05	1.8	yes
cytochrome P450, family 2, subfamily J, polypeptide 2	CYP2J2	Hs.926	205073_at	3.72E-05	1.8	yes
sorcin	SRI	Hs.166011	208921_s_at	3.76E-05	3.3	yes
interleukin enhancer binding factor 2, 45kDa	ILF2	Hs.75117	200052_s_at	3.79E-05	3.0	yes
ribophorin II	RPN2	Hs.501293	208689_s_at	3.79E-05	2.9	yes
NADH dehydrogenase (ubiquinone) flavoprotein 1, 51kDa	NDUFV1	Hs.279518	208714_at	3.82E-05	2.5	yes
fibroblast growth factor (acidic) intracellular binding protein	FIBP	Hs.333417	202041_s_at	3.83E-05	1.8	yes
spastic paraplegia 7, paraplegin (pure and complicated autosomal recessive)	SPG7	Hs.74335	214494_s_at	3.89E-05	1.9	yes
decorin	DCN	Hs.356729	211896_s_at	3.98E-05	0.1	yes*
T-box 2	TBX2	Hs.380964	213417_at	3.99E-05	3.1	yes
ribosomal protein S2	RPS2	Hs.273330	217466_x_at	4E-05	1.4	yes
catechol-O-methyltransferase	COMT	Hs.438720	208817_at	4.02E-05	2.3	yes
myxovirus (influenza virus) resistance 2 (mouse)	MX2	Hs.85266	204994_at	4.05E-05	1.6	yes
nuclear prelamin A recognition factor	NARF	Hs.129895	219862_s_at	4.05E-05	2.2	yes
ATP citrate lyase	ACLY	Hs.120870	201128_s_at	4.06E-05	2.1	yes
capping protein (actin filament) muscle Z-line, beta	CAPZB	Hs.110855	201950_x_at	4.07E-05	2.3	yes
actin, gamma 1	ACTG1	Hs.309763	201550_x_at	4.18E-05	1.8	yes
polymerase (RNA) II (DNA directed) polypeptide H	POLR2H	Hs.426324	209302_at	4.23E-05	2.2	yes
polypyrimidine tract binding protein 1	PTBP1		216306_x_at	4.34E-05	2.4	yes
midkine (neurite growth -promoting factor 2)	MDK	Hs.356729	209035_at	4.37E-05	2.4	yes
