# Supplementary material for: Gene expression profiling of noninvasive primary urothelial tumours using microarrays
Source: Br J Cancer. 2005 Nov 1;93(10):1182–90. doi: 10.1038/sj.bjc.6602813 (PMC2361501; doi:10.1038/sj.bjc.6602813)
Supplement: Supplementary Table 1 Continued-7 [file 93-6602813x8.pdf]

Supplementary table 1. Continued-7.

Gene transcript	Gene symbol	Unigene	Probeset ID	p-value	FC±	Adjusted p<0.05
tumor differentially expressed 1	TDE1	Hs.272168	221473_x_at	4.38E-05	2.3	yes
enolase 1, (alpha)	ENO1	Hs.75140	201231_s_at	4.39E-05	2.3	yes
hypothetical protein FLJ21610	FLJ21610	Hs.29173	219377_at	4.43E-05	2.1	yes
heat shock 90kDa protein 1, beta	HSPCB	Hs.356721	214359_s_at	4.43E-05	3.6	yes
keratin 18	KRT18	Hs.166994	201596_x_at	4.44E-05	2.7	yes
peptidylprolyl isomerase B (cyclophilin B)	PPIB	Hs.273415	200968_s_at	4.46E-05	3.5	yes
small nuclear ribonucleoprotein polypeptide A	SNRPA	Hs.151761	201770_at	4.50E-05	1.5	yes
		Hs.446352	217140_s_at	4.58E-05	3.1	yes
retinoblastoma binding protein 4	RBBP4		217301_x_at	4.84E-05	1.7	yes

±Fold change (median Ta / median N).
*Genes that were not validated (adjusted for multiplicity p<0.05).
