# Supplementary material for: Gene expression profiling of noninvasive primary urothelial tumours using microarrays
Source: Br J Cancer. 2005 Nov 1;93(10):1182–90. doi: 10.1038/sj.bjc.6602813 (PMC2361501; doi:10.1038/sj.bjc.6602813)
Supplement: Supplementary Table 2 [file 93-6602813x9.pdf]

Supplementary table 2. Candidate informative genes that distinguish high grade Ta tumors from PUNLMP/low grade Ta tumors.

Gene transcript	Gene symbol	Unigene	Probeset ID	p-value	FC[#]	Adjusted p<0.05
hypothetical protein FLJ13725	FLJ13725	Hs.152717	45749_at	3.87E-05	0.7	no
cyclin -dependent kinase 4	CDK4	Hs.95577	202246_s_at	4.56E-05	1.4	no
ras homolog gene family, member Q	ARHQ	Hs.442989	212119_at	9.17E-05	1.7	no
diazepam binding inhibitor (GABA receptor modulator, acyl -Coenzyme A binding protein)	DBI	Hs.78888	202428_x_at	1.20E-04	1.9	no
tyrosine 3-monooxygenase/tryptophan 5-monooxygenase activation protein, zeta polypeptide	YWHAZ	Hs.386834	200640_at	1.39E-04	1.6	no
H2A histone family, member Z	H2AFZ	Hs.119192	213911_s_at	2.02E-04	2.4	no
diazepam binding inhibitor (GABA receptor modulator, acyl -Coenzyme A binding protein)	DBI	Hs.78888	211070_x_at	2.42E-04	2.0	no
voltage-dependent anion channel 3	VDAC3	Hs.439253	208845_at	2.46E-04	1.4	no
hypothetical protein MAC30	MAC30	Hs.199695	212282_at	4.50E-04	3.1	no
interleukin enhancer bindin g factor 2, 45kDa	ILF2	Hs.75117	200052_s_at	4.51E-04	1.8	no
likely ortholog of mouse immediate early response, erythropoietin 4	LEREPO4	Hs.368598	201593_s_at	5.11E-04	1.7	no
hypothetical protein MAC30	MAC30	Hs.199695	212281_s_at	5.92E-04	3.6	no
proteasome (prosome, macropain) subunit, beta type, 7	PSMB7	Hs.197071	200786_at	6.85E-04	1.7	no
transgelin 2	TAGLN2	Hs.406504	200916_at	7.64E-04	1.6	no
proteasome (prosome, macropain) subunit, alpha type, 6	PSMA6	Hs.374499	208805_at	8.10E-04	1.4	no
aldo-keto reductase family 1, member A1 (aldehyde reductase)	AKR1A1	Hs.372170	201900_s_at	8.22E-04	1.3	no
peroxiredoxin 1	PRDX1	Hs.180909	208680_at	8.23E-04	1.5	no
chromosome 13 open reading frame 12	C13orf12	Hs.268742	217769_s_at	8.42E-04	1.4	no
jun D proto -oncogene	JUND	Hs.2780	203752_s_at	8.57E-04	1.8	no
formin binding protein 3	FNBP3	Hs.298735	213729_at	8.78E-04	1.9	no
phosphoribosylaminoimidazole carboxylase, phosphoribosylaminoimidazole succinocarboxamide synthetase	PAICS	Hs.444439	201013_s_at	9.78E-04	2.0	no
karyopherin alpha 2 (RAG cohort 1, importin alpha 1)	KPNA2	Hs.252712	211762_s_at	9.79E-04	2.1	no
farnesyl-diphosphate farnesyltransferase 1	FDFT1	Hs.191435	210950_s_at	9.92E-04	1.7	no
microtubule -associated protein, RP/EB family, member 1	MAPRE1	Hs.408754	200713_s_at	1.02E-03	1.3	no
v-erb-b2 erythroblastic leukemia viral oncogene homolog 3 (avian)	ERBB3	Hs.306251	202454_s_at	1.03E-03	2.1	no
diazepam binding inhibitor (GABA receptor modulator, acyl -Coenzyme A binding protein)	DBI	Hs.78888	209389_x_at	1.05E-03	1.8	no
hypothetical protein MAC30	MAC30	Hs.199695	212279_at	1.08E-03	2.5	no
tyrosine 3-monooxygenase/tryptophan 5-monooxygenase activation protein, zeta polypeptide	YWHAZ	Hs.386834	200639_s_at	1.13E-03	1.4	no
exportin 1 (CRM1 homolog, yeast)	XPO1	Hs.157367	208775_at	1.20E-03	2.0	no
