# Supplementary material for: Gene expression profiling of noninvasive primary urothelial tumours using microarrays
Source: Br J Cancer. 2005 Nov 1;93(10):1182–90. doi: 10.1038/sj.bjc.6602813 (PMC2361501; doi:10.1038/sj.bjc.6602813)
Supplement: Supplementary Table 2 Continued-1 [file 93-6602813x10.pdf]

Supplementary table 2. Continued-1.

Gene transcript	Gene symbol	Unigene	Probeset ID	p-value	FC#	Adjusted p<0.05
chromosome 16 open reading frame 34	C16orf34	Hs.437433	212115_at	1.24E-03	1.5	no
phosphatidic acid phosphatase type 2A	PPAP2A	Hs.482121	209147_s_at	1.24E-03	1.7	no
START domain containing 7	STARD7	Hs.445446	200028_s_at	1.35E-03	1.4	no
MCM3 minichromosome maintenance deficient 3 (S. cerevisiae)	MCM3	Hs.179565	201555_at	1.38E-03	1.7	no
slingshot 3	SSH-3	Hs.29173	51192_at	1.41E-03	0.6	no
S-adenosylhomocysteine hydrolase	AHCY	Hs.388004	200903_s_at	1.42E-03	1.8	no
glucose phosphate isomerase	GPI	Hs.406458	208308_s_at	1.45E-03	1.7	no
transferrin receptor (p90, CD71)	TFRC	Hs.185726	208691_at	1.46E-03	3.0	no
small EDRK -rich factor 2	SERF2	Hs.424126	210183_x_at	1.50E-03	1.4	no
RNA -binding protein	FLJ20273	Hs.95549	218035_s_at	1.73E-03	1.7	no
S100 calcium binding protein A2	S100A2	Hs.413843	204268_at	1.78E-03	0.2	no
high-mobility group nucleosomal binding domain 2	HMGN2	Hs.181163	208668_x_at	1.82E-03	1.4	no
ras homolog gene family, member Q	ARHQ	Hs.442989	212117_at	1.90E-03	1.4	no
H2A histone family, member X	H2AFX	Hs.147097	205436_s_at	2.03E-03	2.0	no
thymidylate synthetase	TYMS	Hs.87491	202589_at	2.06E-03	2.4	no
slingshot 3	SSH-3	Hs.29173	219241_x_at	2.07E-03	0.7	no
H2A histone family, member Z	H2AFZ	Hs.119192	200853_at	2.08E-03	1.8	no
chaperonin containing TCP1, subunit 2 (beta)	CCT2	Hs.189772	201947_s_at	2.10E-03	1.4	no
karyopherin (importin) beta 1	KPNB1	Hs.439683	208974_x_at	2.27E-03	1.4	no
claudin 4	CLDN4	Hs.5372	201428_at	2.35E-03	2.0	no
isoleucine-tRNA synthetase	IARS	Hs.172801	204744_s_at	2.45E-03	1.6	no
small nuclear ribonucleoprotein polypeptides B and B1	SNRPB	Hs.83753	208821_at	2.46E-03	1.6	no
bromodomain containing 2	BRD2	Hs.75243	208685_x_at	2.66E-03	1.5	no
mitochondrial ribosomal protein L15	MRPL15	Hs.18349	218027_at	2.69E-03	1.8	no
dynein, axonemal, heavy polypeptide 3	DNAH3	Hs.375739	220725_x_at	2.70E-03	0.4	no
unactive progesterone receptor, 23 kD	TEBP	Hs.355693	200627_at	2.88E-03	1.3	no
heterogeneous nuclear ribonucleoprotein K	HNRPK	Hs.307544	200775_s_at	3.15E-03	1.4	no
chaperonin containing TCP1, subunit 8 (theta)	CCT8	Hs.416211	200873_s_at	3.27E-03	1.7	no
tubulin, alpha, ubiquitous	K-ALPHA-1	Hs.446608	211072_x_at	3.28E-03	2.0	no
cat eye syndrome chromosome region, candidate 1	CECR1	Hs.170310	219505_at	3.35E-03	0.3	no
heterogeneous nuclear ribonucleoprotein R	HNRPR	Hs.15265	208766_s_at	3.37E-03	1.8	no
Homo sapiens cDNA FLJ12835 fis, clone NT2RP2003165.		Hs.388918	216153_x_at	3.39E-03	0.4	no
HSPC038 protein	LOC51123	Hs.449945	218059_at	3.42E-03	1.8	no
tyrosine 3-monooxygenase/tryptophan 5-monooxygenase activation protein, theta polypeptide	YWHAQ	Hs.74405	212426_s_at	3.44E-03	1.4	no
tubulin, beta, 4	TUBB4	Hs.511743	213476_x_at	3.50E-03	1.5	no
guanine nucleotide binding protein (G protein), alpha inhibiting activity polypeptide 2	GNAI2	Hs.77269	201040_at	3.56E-03	0.8	no
high-mobility group box 2	HMGB2	Hs.434953	208808_s_at	3.71E-03	3.9	no
oligophrenin 1	OPHN1	Hs.128824	206323_x_at	3.84E-03	0.4	no
keratin 13	KRT13	Hs.433871	207935_s_at	3.89E-03	0.3	no
mitochondrial ribosomal protein L3	MRPL3	Hs.320961	208787_at	3.96E-03	1.6	no
