# Supplementary material for: Gene expression profiling of noninvasive primary urothelial tumours using microarrays
Source: Br J Cancer. 2005 Nov 1;93(10):1182–90. doi: 10.1038/sj.bjc.6602813 (PMC2361501; doi:10.1038/sj.bjc.6602813)
Supplement: Supplementary Table 2 Continued-2 [file 93-6602813x11.pdf]

Supplementary table 2. Continued-2.

Gene transcript	Gene symbol	Unigene	Probeset ID	p-value	FC#	Adjusted p<0.05
tyrosine 3-monooxygenase/tryptophan 5-monooxygenase activation protein, zeta polypeptide	YWHAZ	Hs.386834	200638_s_at	4.00E-03	1.9	no
nucleolar protein fam ily A, member 3 (H/ACA small nucleolar RNPs)	NOLA3	Hs.14317	217962_at	4.06E-03	1.3	no
chaperonin containing TCP1, subunit 2 (beta)	CCT2	Hs.189772	201946_s_at	4.09E-03	2.0	no
tubulin, alpha, ubiquitous	K-ALPHA -1	Hs.446608	213646_x_at	4.17E-03	1.9	no
nucleostemin	NS	Hs.313544	217850_at	4.24E-03	1.6	no
tyrosine 3-monooxygenase/tryptophan 5-monooxygenase activation protein, theta polypeptide	YWHAQ	Hs.74405	200693_at	4.26E-03	1.3	no
heterogeneous nuclear ribonucleoprotein A/B	HNRPAB	Hs.81361	201277_s_at	4.28E-03	1.3	no
proliferating cell nuclear antigen	PCNA	Hs.78996	201202_at	4.31E-03	2.4	no
CSE1 chromosome segregation 1-like (yeast)	CSE1L	Hs.90073	201112_s_at	4.33E-03	1.6	no
Homo sapiens transcribed sequences		Hs.74405	213699_s_at	4.34E-03	1.4	no
MCM5 minichromosome maintenance deficient 5, cell division cycle 46 (S. cerevisiae)	MCM5	Hs.77171	216237_s_at	4.37E-03	2.3	no
hypothetical protein DKFZp434K1210	DKFZp434K1210	Hs.32352	218149_s_at	4.39E-03	1.4	no
fatty acid binding protein 4, adipocyte	FABP4	Hs.391561	203980_at	4.41E-03	0.1	no
tubulin, beta, 2	TUBB2	Hs.433615	208977_x_at	4.51E-03	1.5	no
procollagen-proline, 2-oxoglutarate 4-dioxygenase (proline 4-hydroxylase), beta polypeptide (protein disulfide isomerase; thyroid hormone binding protein p55)	P4HB	Hs.410578	200656_s_at	4.61E-03	1.5	no
malate dehydrogenase 1, NAD (soluble)	MDH1	Hs.75375	200978_at	4.96E-03	1.4	no
solute carrier family 16 (monocarboxylic acid transporters), member 3	SLC16A3	Hs.8752	202857_at	5.00E-03	1.4	no

Fold change (median high grade/median PUNLMP/low grade)
