# Supplementary material for: Gene expression profiling of noninvasive primary urothelial tumours using microarrays
Source: Br J Cancer. 2005 Nov 1;93(10):1182–90. doi: 10.1038/sj.bjc.6602813 (PMC2361501; doi:10.1038/sj.bjc.6602813)
Supplement: Supplementary Table 3 [file 93-6602813x12.pdf]

**Supplementary table 3.** Protein expression of KRT7, SDC1, CDK4, and JUND in normal bladder and Ta tumor.

| | | | NORMAL | | | | TUMOR | | | |
|---|---|---|---|---|---|---|---|---|---|---|
| | | | urothelial cells | | stroma cells | | cancer cells | | stroma cells | |
| Sample | Tissue type | Epitope | Nuclei (%) | Cytoplasma (%) | Nuclei (%) | Cytoplasma (%) | Nuclei (%) | Cytoplasma (%) | Nuclei (%) | Cytoplasma (%) |
| N1 | Normal bladder | KRT7 | 0 | 100 | 0 | 0 | | | | |
| N2 | Normal bladder | KRT7 | 0 | 80 | 0 | 0 | | | | |
| N3 | Normal bladder | KRT7 | 0 | 80 | 0 | 0 | | | | |
| N4 | Normal bladder | KRT7 | 0 | 90 | 0 | 0 | | | | |
| N5 | Normal bladder | KRT7 | 0 | 100 | 0 | 0 | | | | |
| N6 | Normal bladder | KRT7 | 0 | 80 | 0 | 0 | | | | |
| **Median (range)** | | | **0** | **85 (80-100)** | **0** | **0** | | | | |
| T1 | Ta | KRT7 | | | | | 0 | 100 | 0 | 0 |
| T2 | Ta | KRT7 | | | | | 0 | 100 | 0 | 0 |
| T3 | Ta | KRT7 | | | | | 0 | 100 | 0 | 0 |
| T4 | Ta | KRT7 | | | | | 0 | 100 | 0 | 0 |
| T5 | Ta | KRT7 | | | | | 0 | 100 | 0 | 0 |
| **Median/ range** | | | | | | | **0** | **100** | **0** | **0** |
| N1 | Normal bladder | SDC1 | 0 | 10 | 0 | 0 | | | | |
| N2 | Normal bladder | SDC1 | 0 | 25 | 0 | 0 | | | | |
| N3 | Normal bladder | SDC1 | 0 | 80 | 10 | 10 | | | | |
| N4 | Normal bladder | SDC1 | 0 | 80 | 0 | 0 | | | | |
| N5 | Normal bladder | SDC1 | 0 | 100 | 25 | 0 | | | | |
| N6 | Normal bladder | SDC1 | 0 | 50 | 0 | 0 | | | | |
| **Median/ range** | | | **0** | **65 (10-100)** | **0 (0-25)** | **0 (0-10)** | | | | |
| T1 | Ta | SDC1 | | | | | 0 | 100 | 0 | 0 |
| T2 | Ta | SDC1 | | | | | 0 | 90 | 0 | 0 |
| T3 | Ta | SDC1 | | | | | 0 | 100 | 0 | 0 |
| T4 | Ta | SDC1 | | | | | 0 | 100 | 0 | 0 |
| T6 | Ta | SDC1 | | | | | 0 | 100 | 0 | 0 |
| **Median/ range** | | | | | | | **0** | **100 (90-100)** | **0** | **0** |
| N1 | Normal bladder | CDK4 | 5 | 5 | 50 | 50 | | | | |
| N2 | Normal bladder | CDK4 | 5 | 30 | 50 | 50 | | | | |
| N3 | Normal bladder | CDK4 | 1 | 10 | 50 | 50 | | | | |
| N4 | Normal bladder | CDK4 | 10 | 60 | 30 | 10 | | | | |
| **Median (range)** | | | **5 (1-10)** | **20 (5-60)** | **50 (30-50)** | **50 (10-50)** | | | | |
| T1 | Ta | CDK4 | | | | | 10 | 100 | 10 | 30 |
| T3 | Ta | CDK4 | | | | | 50 | 100 | 50 | 50 |
| T4 | Ta | CDK4 | | | | | 10 | 100 | 50 | 50 |
| T7 | Ta | CDK4 | | | | | 100 | 100 | 10 | 10 |
| T8 | Ta | CDK4 | | | | | 100 | 100 | 0 | 0 |
| **Median (range)** | | | | | | | **50 (10-100)** | **100** | **10 (0-50)** | **30 (0-50)** |
| N1 | Normal bladder | JUND | 0 | 0 | 0 | 0 | | | | |
| N2 | Normal bladder | JUND | 10 | 30 | 50 | 10 | | | | |
| N3 | Normal bladder | JUND | 100 | 100 | 10 | 10 | | | | |
| N4 | Normal bladder | JUND | 100 | 100 | 50 | 10 | | | | |
| **Median/ range** | | | **55 (0-100)** | **65 (0-100)** | **30 (0-50)** | **10 (0-10)** | | | | |
| T9 | Ta | JUND | | | | | 100 | 100 | 10 | 0 |
| T3 | Ta | JUND | | | | | 50 | 80 | 50 | 0 |
| T4 | Ta | JUND | | | | | 80 | 100 | 30 | 10 |
| T7 | Ta | JUND | | | | | 100 | 80 | 10 | 10 |
| T8 | Ta | JUND | | | | | 100 | 100 | 10 | 0 |
| T10 | Ta | JUND | | | | | 50 | 100 | 50 | 50 |
| **Median/ range** | | | | | | | **90 (50-100)** | **100 (80-100)** | **20 (10-50)** | **5 (0-50)** |
